# Acyl-CoA Thioesterase 8 and 11 as Novel Biomarkers for Clear Cell Renal Cell Carcinoma

**DOI:** 10.3389/fgene.2020.594969

**Published:** 2020-12-10

**Authors:** Chao-Liang Xu, Lei Chen, Deng Li, Fei-Teng Chen, Ming-Lei Sha, Yi Shao

**Affiliations:** ^1^Department of Urology, Shanghai General Hospital, Shanghai Jiao Tong University School of Medicine, Shanghai, China; ^2^Department of Geriatric, Shanghai General Hospital, Shanghai Jiao Tong University School of Medicine, Shanghai, China

**Keywords:** ACOT8, ACOT11, clear cell renal cell carcinoma, biomarkers, oxidative phosphorylation, ferroptosis

## Abstract

**Background:**

Clear cell renal cell carcinoma (ccRCC) is essentially a metabolic disorder characterized by reprogramming of several metabolic pathways. Acyl-coenzyme A thioesterases (ACOTs) are critical enzymes involved in fatty acid metabolism; however, the roles of ACOTs in ccRCC remain unclear. This study explored ACOTs expressions and their diagnostic and prognostic values in ccRCC.

**Methods:**

Three online ccRCC datasets from The Cancer Genome Atlas (TCGA) and the Gene Expression Omnibus (GEO) were utilized to measure the expressions of *ACOTs* in paired normal and tumor tissues. Receiver operating characteristic (ROC) curves were depicted to assess the diagnostic values of *ACOTs* in ccRCC. Quantitative real-time PCR and immunohistochemical analysis were performed to validate the ACOT11 expression in ccRCC cell lines and clinical samples. Survival curves and Cox regression analysis were used to evaluate the predictive values of *ACOTs* in clinical outcome of ccRCC patients. Functional enrichment analyses and correlation analysis were carried out to predict the potential roles of ACOT8 in tumorigenesis and progression of ccRCC.

**Results:**

*ACOT1/2/8/11/13* were found to be significantly downregulated in ccRCC samples. In particular, *ACOT11* was decreased in almost every matched normal-tumor pair, and had extremely high diagnostic value as shown by ROC curve analysis (AUC = 0.964). The expression of ACOT11 was further verified in ccRCC cell lines and clinical samples at mRNA and protein levels. Furthermore, clinical correlation analysis and survival analysis indicated that *ACOT8* was correlated with disease progression and was an independent predictor of unfavorable outcome in ccRCC. Moreover, functional analyses suggested potential roles of ACOT8 in the regulation of oxidative phosphorylation (OXPHOS), and correlation analysis revealed an association between *ACOT8* and ferroptosis-related genes in ccRCC.

**Conclusion:**

Our study revealed that ACOT11 and ACOT8 are promising biomarkers for diagnosis and prognosis of ccRCC, respectively, and ACOT8 may affect ccRCC development and progression through the regulation of OXPHOS and ferroptosis. These findings may provide new strategies for precise diagnosis and personalized therapy of ccRCC.

## Introduction

In the United States, kidney cancer is estimated to be the sixth most common form of cancer in men and the eighth in women in 2020, accounting for approximately 5 and 3% of all newly diagnosed cancers, respectively (Siegel et al., 2020). Renal cell carcinoma (RCC) represents 85% of all kidney cancers, and its incidence has been continuously increasing in the past two decades (Capitanio and Montorsi, 2016; Barata and Rini, 2017). Although surgical excision improves the 5-year survival rate for early-stage RCC patients, it is hard to put a brake on cancer progression and death of patients present with metastasis at the time of diagnosis, which account for up to one-third of all patients (Patard et al., 2011). In addition, RCC is often discovered incidentally through imaging examination for unrelated symptoms (Roupret et al., 2018). Given that clear cell renal cell carcinoma (ccRCC) is the most common subtype of RCC (Barata and Rini, 2017), a highly effective molecular biomarker for ccRCC that can be widely accepted is urgently needed.

Clear cell renal cell carcinoma essentially acts as a metabolic disorder with *VHL* mutations occurring in approximately 90% of patients and is characterized by reprogramming of several metabolic pathways (Nickerson et al., 2008). The reprogramming of glucose metabolism, fatty acid metabolism, oxidative phosphorylation (OXPHOS), and amino acid metabolism such as glutamine, arginine and tryptophan have pivotal regulatory roles in development and progression of ccRCC, enabling cancer cells to thrive in the hypoxic and auxotrophic microenvironment (Wettersten et al., 2017). Among these metabolic pathways, augmented lipogenesis is one of the most significant events in ccRCC for its central roles in membrane formation, cellular signaling, and cell proliferation (Lucarelli et al., 2019). Consistently, studies also indicated that long-chain fatty acids increased (Hakimi et al., 2016) and enzymes involved in β-oxidation decreased in ccRCC tissues through metabolomics and proteomics analysis, respectively (Wettersten et al., 2015). Accordingly, enzymes involved in fatty acid metabolism might be promising biomarkers indicative of diagnosis and prognosis in ccRCC.

As an essential enzyme family in mammalian fatty acid metabolism, acyl-coenzyme A thioesterases (ACOTs) catalyze the hydrolysis reaction of fatty acyl-CoA ester to release coenzyme A and the corresponding non-esterified fatty acid, thereby regulating β-oxidation, lipid biosynthesis, signal transduction, and several important cellular processes (Kirkby et al., 2010). To date, 10 ACOT family members have been identified in human genomes. All of them are grouped in two types on the basis of molecular weight with high sequence conservation within each type: type-I (includes ACOT1, ACOT2, ACOT4, and ACOT6) consisting of an N-terminal β-sandwich domain and a C-terminal α/β hydrolase domain and type-II (includes ACOT7, ACOT8, ACOT9, ACOT11, ACOT12, and ACOT13) containing one or more copies of the hotdog domain (Kirkby et al., 2010).

Several studies have demonstrated that some members of ACOTs are aberrantly expressed and exert different roles in multiple malignancies. ACOT8 have been observed to promote the growth of hepatocellular carcinoma cell lines via releasing free fatty acid to meet energy requirement (Hung et al., 2014). Moreover, a recent study showed that higher ACOT7 expression was associated with worse overall survival in breast and lung cancer patients (Jung et al., 2017). Even if most ACOTs have broad expression in kidney according to NCBI Gene database, it is not known yet whether they are implicated in kidney cancer.

In the present study, we evaluated the diagnostic and prognostic value of eight ACOT family members in ccRCC patients, while ACOT6 and ACOT12 were excluded owing to extremely low expression in kidney. Finally, we identified ACOT11 as a diagnostic marker and ACOT8 as a prognostic marker for ccRCC. Further functional analysis and correlation analysis revealed possible roles of ACOT8 in the regulation of OXPHOS and ferroptosis, thus providing new opportunities for therapy of ccRCC.

## Materials and Methods

### UALCAN

UALCAN^1^ is a common useful website for online statistical analysis and data mining based on RNA-seq and clinical data in TCGA database, providing the transcriptional expression levels of genes of interest in normal and tumor samples and their association with clinicopathologic parameters (Chandrashekar et al., 2017). In this study, UALCAN was utilized to show the mRNA expression levels of *ACOTs* in ccRCC and normal kidney samples, where *p*-value < 0.05 was statistically significant. Furthermore, a list of *ACOT8* co-expressed genes in TCGA KIRC dataset was obtained from UALCAN, and absolute value of correlation coefficient |*r*| > 0.35 served as the threshold for statistical significance.

### The Cancer Genome Atlas Database

The Cancer Genome Atlas (TCGA)^2^ is a landmark cancer genomics project which provides sequencing and clinicopathologic information for 33 kinds of human cancer currently (Tomczak et al., 2015). In our study, mRNA expression data of 526 ccRCC and 72 normal kidney samples determined by the value of Fragments Per Kilobase per Million, and clinical data of 512 ccRCC patients were downloaded from TCGA database. Among these 598 samples, there were 71 paired ccRCC and adjacent normal kidney samples. Twenty-five ccRCC samples were excluded due to the absence of complete clinical information when clinical correlation analysis and survival analysis were performed.

### The Gene Expression Omnibus Database

Gene Expression Omnibus (GEO)^3^ is a well-known database that collects and processes high-throughput genomic data uploaded by global researchers, including microarray chips, next-generation sequencing, and other forms of data (Edgar et al., 2002; Barrett et al., 2013). In this study, the transcriptional expression data were downloaded from the datasets of GSE53757 and GSE40435 in the GEO database, containing 72 and 101 paired ccRCC and normal kidney specimens, respectively.

### Cell Culture

The normal human tubular epithelial cell line HK-2 and ccRCC cell lines (786-O and 769-P) were all obtained from the Cell Bank of the Chinese Academy of Sciences (Shanghai, China) and grown in Dulbecco’s modified Eagle’s medium (DMEM, Gibco) and RPMI 1640 medium (Hyclone), respectively. The culture medium was supplemented with 10% fetal bovine serum (FBS, Gibco) and 1% penicillin/streptomycin (Gibco). The cells were incubated at 37°C in a humidified atmosphere containing 5% CO_2_.

### Quantitative Real-Time PCR

Total RNA was extracted from cell lines and clinical samples using RNA-Quick Purification Kit (Yishan, Shanghai, China) according to the manufacturer’s specification. mRNA was reverse-transcribed into cDNA using PrimeScript RT Master Mix (Takara). To determine the mRNA expressions of different *ACOTs*, quantitative real-time PCR (qRT-PCR) was performed with SYBR Premix Ex Taq II (Takara) in a QuantStudio 7 Flex Real-Time PCR System (Applied Biosystems). The comparative CT method (2^–ΔΔ*CT*^) was used to calculate the relative expression levels of each target gene with β-actin as an internal control. The specific primer sequences used are listed in Supplementary Table 1.

### Clinical Samples and Immunohistochemical Staining

Clear cell renal cell carcinoma and adjacent normal kidney samples were collected from 15 patients without chemotherapy or radiotherapy to validate the expression change of ACOT11 in ccRCC. Our study was approved by the ethics committee of our hospital, and all these patients signed an informed consent form. The paraffin-embedded tissue sections were prepared for immunohistochemical staining according to standard procedures. Then, a rabbit polyclonal antibody to human ACOT11 (ab153835) was applied for the detection of ACOT11 expression in clinical samples.

### Online Consensus Survival Analysis for KIRC

Online consensus survival analysis for KIRC (OSkirc)^4^ is a free web tool that collects a total of 629 ccRCC cases with gene expression data and clinical follow-up information from TCGA and the GEO databases (GSE22541, GSE29609, and GSE3) and can be used for survival analysis of interesting genes (Xie et al., 2019). In our study, OSkirc was utilized to verify the survival significance of *ACOTs* in ccRCC, where *p*-value < 0.05 was statistically significant.

### Gene Ontology and Kyoto Encyclopedia of Genes and Genomes Enrichment Analysis

Gene Ontology (GO) analysis, comprising biological process (BP), molecular function (MF), and cellular component (CC), as well as Kyoto Encyclopedia of Genes and Genomes (KEGG) pathway analysis, are common methods for functional annotation and enrichment analysis of gene and gene clusters. In this study, GO and KEGG pathway analysis in The Database for Annotation, Visualization, and Integrated Discovery (DAVID)^5^ were performed on genes co-expressed with *ACOT8* in TCGA KIRC dataset (Huang et al., 2009; Huang da et al., 2009). *p-*Value < 0.05 and false discovery rate (FDR) < 0.05 were regarded to be statistically significant.

### Protein–Protein Interactions Network Analysis

STRING^6^, which is an online database to search for known proteins and generate protein–protein interactions (PPI) network, was utilized to analyze *ACOT8* co-expressed genes in TCGA KIRC dataset (Szklarczyk et al., 2017, 2019). The PPI network was screened with the highest confidence score (>0.900), and the disconnected nodes were excluded out of network. To further assess the biological function of ACOT8 in ccRCC, the processed PPI network was input into Cytoscape 3.8.0 software, and the hub genes with the highest score were selected out by using the MCODE plug-in.

### Gene Set Enrichment Analysis

Gene set enrichment analysis (GSEA) is a powerful tool that can identify upregulated genes related to disease phenotypes among a large number of genes, thus can be used to judge the relationship between the disease phenotypes and the genes of interest (Mootha et al., 2003; Subramanian et al., 2005). The ccRCC samples from TCGA database were divided into two groups based on the median of transcriptional expression of *ACOT8* because the expression value disobeyed the Gaussian distribution. Then, GSEA software was applied to compare the expression profiles between these two groups. A nominal *p-*value < 0.05 and FDR < 0.25 were regarded as a significantly enriched gene set.

### Statistical Analysis

SPSS software version 20.0 was used to carry out statistical analysis and calculate the area under the curve (AUC) score of ROC curve. Wilcoxon matched-pairs signed rank test was implemented to examine the significant differences between paired ccRCC and adjacent normal kidney samples. The potential association between *ACOTs* and clinicopathologic parameters was analyzed by Pearson Chi-squared test. Graphpad Prism 7 was applied to generate survival curves, and Log-rank test was used to determine the significant differences. Univariate and multivariate Cox regression analyses were carried out to further assess the prognostic value of *ACOTs* for ccRCC. *ACOTs* expressions were entered as categorical variables according to their respective median values. Factors with *p-*value < 0.1 in the univariate Cox analysis were incorporated into the subsequent multivariate Cox analysis. Only *p-*value < 0.05 was considered as statistically significant.

## Results

### Different *ACOTs* Are Low Expressed in ccRCC Patients From TCGA and GEO Databases

**FIGURE 1:**
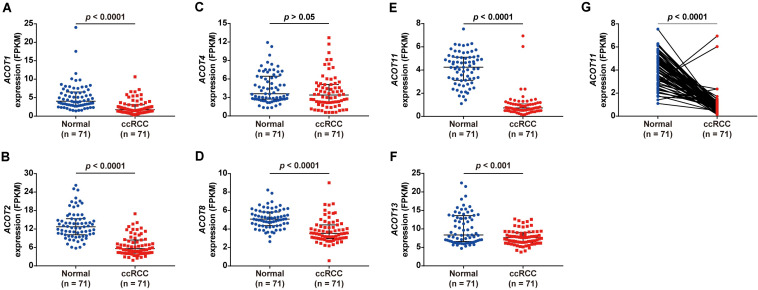
Different *ACOTs* expressions in 71 ccRCC normal-tumor pairs from TCGA database. *ACOT1/2/8/11/13* were significantly downregulated in ccRCC, while the transcriptional expression of *ACOT4* was not significantly different between these two groups **(A–F)**. *ACOT11* expression obviously decreased in most matched samples **(G)**. *p-*Value < 0.05 was considered as statistically significant.

To explore the diagnostic and prognostic value of different *ACOTs* in ccRCC patients, their transcriptional expression patterns were initially analyzed by UALCAN. As shown in Supplementary Figure 1, mRNA expressions of *ACOT1/2/4/8/11/13* in ccRCC were significantly downregulated compared with that in normal kidney samples, while the expressions of *ACOT7/9* were not significantly different between these two groups. To further verify the expression changes of *ACOTs* in ccRCC, the expression data of 71 paired ccRCC and adjacent normal kidney samples were downloaded from TCGA database. As shown in Figure 1, *ACOT1/2/8/11/13* were significantly low expressed in ccRCC samples, which was consistent with the analysis result from UALCAN. However, *ACOT4* expression in ccRCC was almost the same as that in matched normal kidney samples. Moreover, two datasets downloaded from the GEO database (GSE40435 and GSE53757) that contained 101 and 72 paired ccRCC and normal kidney samples, respectively, further confirmed that *ACOT1/2/8/11/13* were significantly downregulated in ccRCC (Supplementary Figures 2, 3). Interestingly, it was noticeable that *ACOT11* was markedly downregulated in almost every matched specimen in both TCGA and GEO databases, suggesting that ACOT11 may be a potential diagnostic marker for ccRCC (Figure 1G and Supplementary Figures 2F, 3E). Altogether, these results indicate that ACOT1/2/8/11/13 may play roles in tumorigenesis of ccRCC.

### Diagnostic Value of mRNA Expressions of *ACOTs* in ccRCC Patients

**FIGURE 2:**
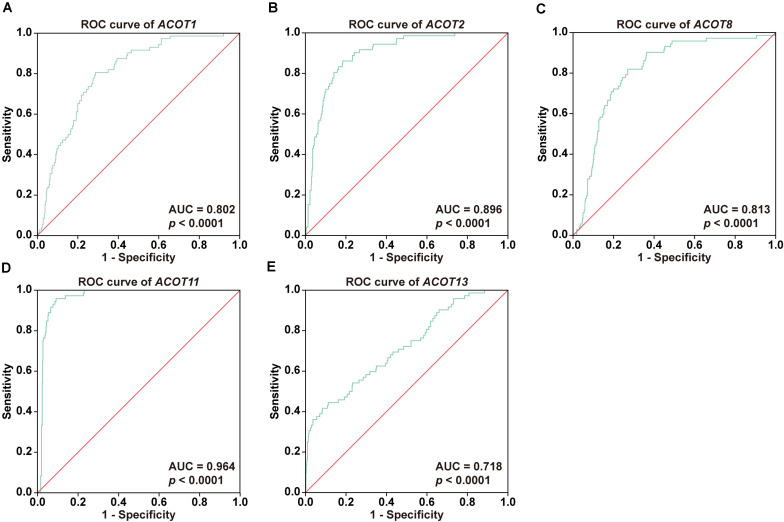
Diagnostic values of different *ACOTs* in ccRCC were shown by ROC curves. The transcriptional expression data of *ACOT1/2/8/11/13* in 526 ccRCC and 72 normal kidney samples from TCGA KIRC dataset were used in ROC analysis. ROC curves and respective AUC scores are shown in **(A–E)**. *p-*Value < 0.05 was regarded as statistically significant.

**FIGURE 3:**
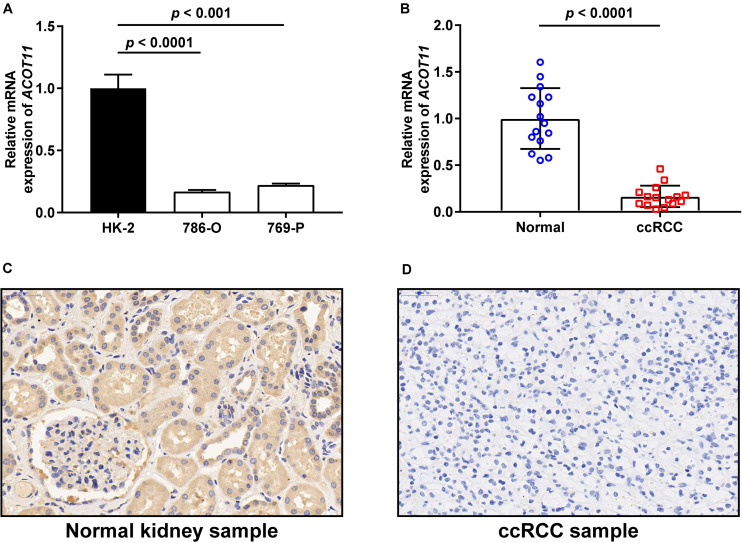
Validation of ACOT11 expression in ccRCC by *in vitro* and *in vivo* experiments. The mRNA expression of *ACOT11* in ccRCC were verified in cell lines **(A)** and clinical samples **(B)** by qRT-PCR. The protein level of ACOT11 in ccRCC were verified in clinical samples by immunohistochemistry staining **(C,D)**. *p-*Value < 0.05 was considered to be statistically significant.

To further investigate the diagnostic value of *ACOTs* in ccRCC patients, transcriptional expression data of 598 samples (72 normal vs 526 tumor) from TCGA database were utilized to perform receiver operating characteristic (ROC) analysis, and the AUC was calculated to quantify the diagnostic value of *ACOTs*. The results showed that the AUC scores for *ACOT1/2/8/11/13* were all greater than 0.7, suggesting a certain value in discriminating ccRCC patients (Figure 2). Remarkably, the AUC score of *ACOT11* was 0.964, which exceeded that of other *ACOT* family members. Given that *ACOT11* showed fairly consistent expression changes in matched samples from TCGA and GEO databases, we speculated that ACOT11 might be a potential diagnostic marker for ccRCC with extremely high specificity and sensitivity. The expression pattern of ACOT11 was further verified in cell lines and clinical samples. qRT-PCR analysis indicated that *ACOT11* mRNA expression was markedly reduced in ccRCC cell lines 786-O and 769-P, compared with that in normal human tubular epithelial cell line HK-2 (Figure 3A). Similarly, in contrast with adjacent normal kidney, the mRNA level of *ACOT11* was decreased in ccRCC samples (Figure 3B). Furthermore, immunohistochemistry also revealed low protein expression of ACOT11 in ccRCC samples (Figures 3C,D). Hence, all these results suggest ACOT11 could serve as a promising diagnostic biomarker for ccRCC. In addition, the downregulation of *ACOT1/8/13* in ccRCC was also confirmed by qRT-PCR, whereas *ACOT2* showed inconsistent expression changes *in vitro* and *in vivo* (Supplementary Figure 4).

### Association of *ACOTs* With Clinicopathologic Parameters of ccRCC Patients

**FIGURE 4:**
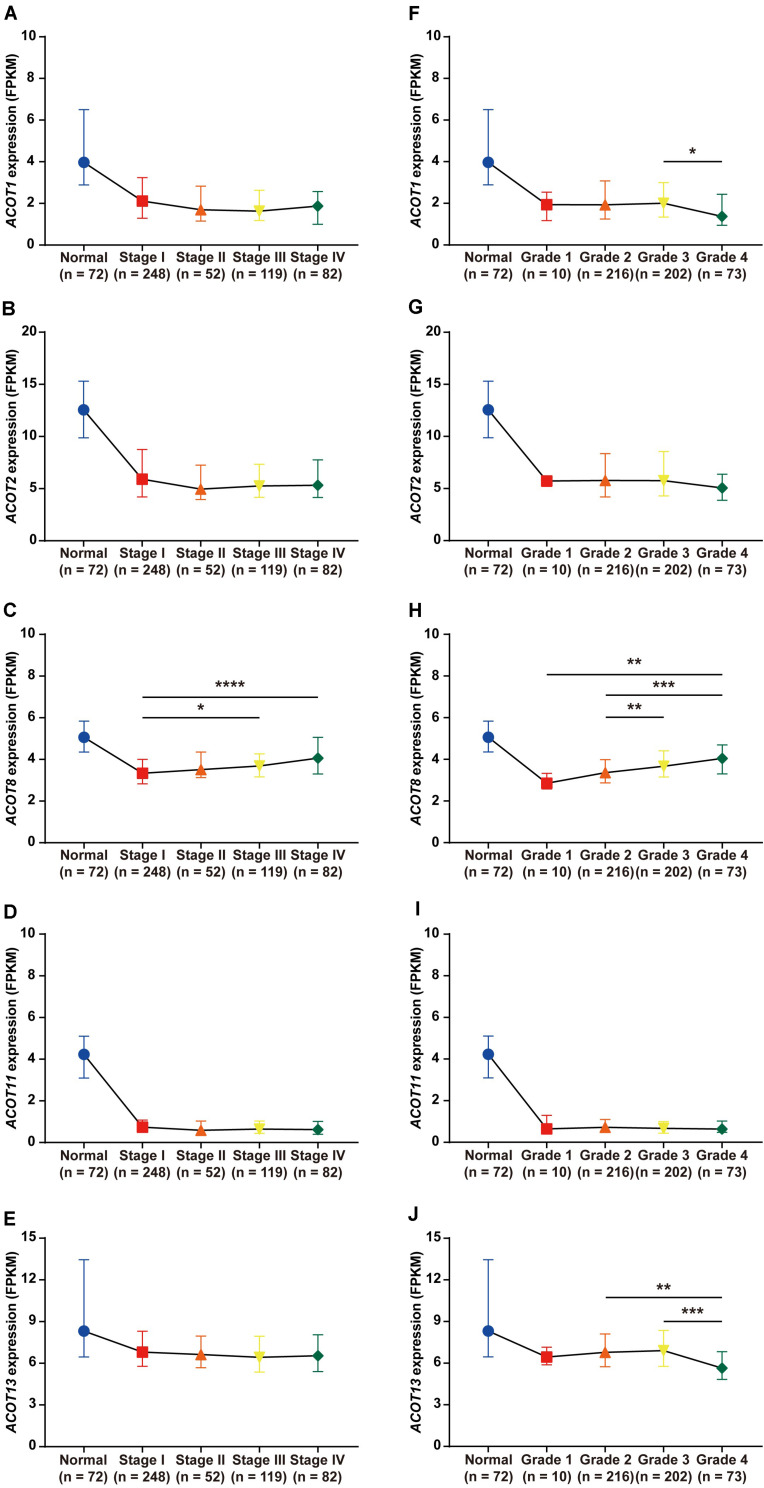
Relation between different *ACOTs* expressions and disease progression in ccRCC patients. ccRCC and normal kidney samples with complete clinical data were utilized to analyze the relationship between mRNA expressions of *ACOTs* and TNM stages **(A–E)** as well as histological grades **(F–J)**. There was no obvious correlation between *ACOT1/2/11/13* expressions and tumor progression, while *ACOT8* was continuously upregulated with tumor progression. *p < 0.05, **p < 0.01, ***p < 0.001, ****p < 0.0001.

Next, we focused on the relationship between the transcriptional expressions of *ACOT1/2/8/11/13* and clinicopathologic parameters of ccRCC patients from TCGA database. Twenty-five ccRCC samples were excluded due to lack of complete clinical information, and the remaining 501 ccRCC and 72 normal kidney samples were included for further analysis. First, we analyzed the mRNA expression patterns of five *ACOTs* in different TNM stages. As was shown in Figures 4A–E, the expression levels of *ACOTs* at stage I were obviously lower than that in the normal group. Intriguingly, the mRNA expressions of *ACOT1/2/11/13* seemed not to be significantly associated with the increase in TNM stages, whereas patients who were in more advanced TNM stages had a tendency to express higher *ACOT8*. Next, we analyzed *ACOTs* expressions in distinct histological grades of ccRCC (Figures 4F–J). Similarly, there was no significant relation between *ACOT1/2/11/13* and histological grades, while the mRNA level of *ACOT8* increased with tumor grades. Furthermore, we performed clinical correlation analysis in these 501 ccRCC samples (Table 1 and Supplementary Table 2). We divided them into high-expression group and low-expression group based on the median of the mRNA levels of these five *ACOTs*, respectively, and selected age, gender, TNM stage, and histological grade as the clinicopathologic parameters to be analyzed. The result indicated that there was no correlation between *ACOTs* expression levels and age, but *ACOT2/11/13* expressions tended to be higher in female patients. Importantly, *ACOT8/11* had a significant correlation with TNM stages, but only *ACOT8* expression was closely related to histological grades. Taken together, these results demonstrate that the transcriptional expression of *ACOT8* is significantly associated with tumor progression in ccRCC patients.

**TABLE 1:** Association of different *ACOTs* with clinicopathologic characteristics in ccRCC patients.

Classification	Number of cases	*ACOT8*		*p*-Value	*ACOT11*		*p*-Value
				
		Low (*n* = 250)	High (*n* = 251)		Low (*n* = 250)	High (*n* = 251)	
**Age (year)**							
<60	231 (46.1%)	112	119	0.558	119	112	0.504
≥60	270 (53.9%)	138	132		131	139	
**Gender**							
Male	329 (65.7%)	168	161	0.471	175	154	*0.042*
Female	172 (34.3%)	82	90		75	97	
**TNM stage**							
I + II	300 (59.9%)	174	126	<*0.000*	136	164	*0.013*
III + IV	201 (40.1%)	76	125		114	87	
**Histological grade**							
G1–2	226 (45.1%)	137	89	<*0.000*	107	119	0.300
G3–4	275 (54.9%)	113	162		143	132	

*p-Value less than 0.05 are in italics.*

### Prognostic Value of Different *ACOTs* in ccRCC Patients

**FIGURE 5:**
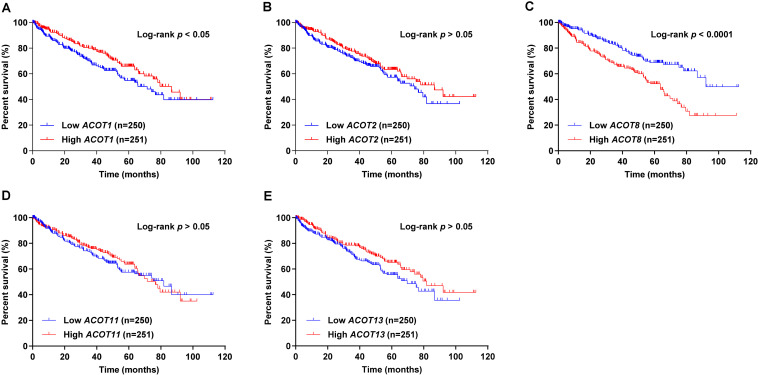
Survival analysis of different ACOTs in ccRCC. ccRCC samples with complete clinical data from TCGA KIRC dataset were used for survival analysis of different *ACOTs*. *ACOT1/8* expressions were significantly associated with the overall survival of ccRCC patients **(A,C)**, while the mRNA expressions of *ACOT2/11/13* showed no correlation with the prognosis of ccRCC patients **(B,D,E)**. Log-rank *p-*value < 0.05 was considered to be statistically significant.

Next, we used survival curves to further evaluate the prognostic value of different *ACOTs* in ccRCC patients. We included 501 ccRCC samples from TCGA database in the survival analysis, and used the median of expression values of each *ACOTs* as the cut-off for grouping. As shown in Figure 5, *ACOT2/11/13* showed no significant correlation with the prognosis of ccRCC patients. Notably, higher expression of *ACOT1* was related to longer overall survival (Figure 5A), whereas higher *ACOT8* expression was significantly associated with unfavorable outcome in ccRCC (Figure 5C). These results were further validated by OSkirc, a web tool which includes a total of 629 ccRCC cases from TCGA and the GEO databases (Supplementary Figure 5). To assess whether *ACOT1/8* were independent prognostic factors for ccRCC, we performed Cox regression survival analysis in the next step. The result of univariate Cox analysis indicated that older age, higher TNM stage, and histological grade, lower *ACOT1* and higher *ACOT8* expression were significantly related to worse prognosis of ccRCC patients (Table 2 and Supplementary Table 3). Remarkably, multivariate Cox analysis revealed that *ACOT1* expression was not an independent prognostic factor for ccRCC, while mRNA expression of *ACOT8* was independently associated with the prognosis of ccRCC patients. In conclusion, all these results indicate that ACOT8 is the only one of ACOT family members with predictive value, and thus could be a potential prognostic marker for ccRCC.

**TABLE 2:** Univariate and multivariate Cox regression analyses of overall survival in ccRCC patients.

Risk factors	Univariate analysis			Multivariate analysis		
		
	HR	95% CI	*p*-Value	HR	95% CI	*p*-Value
Age	1.804	1.291–2.521	*0.001*	1.647	1.173–2.311	*0.004*
Gender	1.061	0.764–1.474	0.723			
TNM stage	4.241	3.006–5.982	*0.000*	3.120	2.172–4.482	*0.000*
Histological grade	2.826	1.945–4.107	*0.000*	1.882	1.275–2.779	*0.001*
*ACOT8* expression	2.001	1.439–2.782	*0.000*	1.634	1.164–2.294	*0.005*

*p-Value less than 0.05 are in italics.*

### Functional Analysis of ACOT8 in ccRCC

**FIGURE 6:**
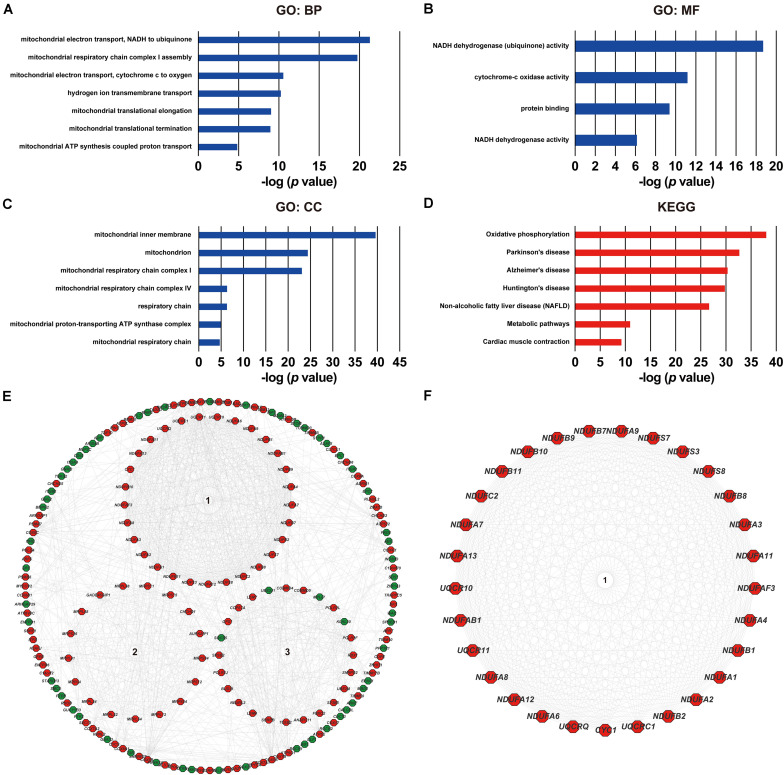
Functional analyses of ACOT8 by GO, KEGG pathway and PPI network. GO functional enrichment analysis comprising biological process (BP, **A**), molecular function (MF, **B**) and cellular component (CC, **C**), as well as KEGG pathway analysis **(D)** were performed on *ACOT8* co-expressed genes in TCGA KIRC dataset. These co-expressed genes were further utilized to construct a PPI network by Cytoscape software, and the top three hub gene clusters are shown in the form of circle in the middle of **(E)**. Positive-related genes are shown in red, while negative-related genes in green. The gene cluster with the highest score of connectivity is separately presented in **(F)**.

**FIGURE 7:**
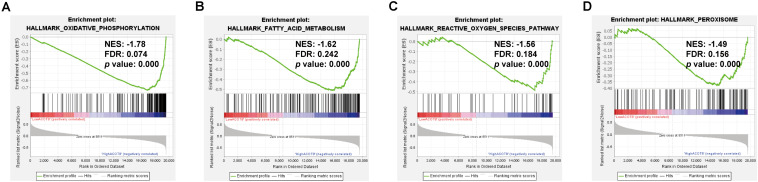
Significantly enriched gene sets in high *ACOT8* samples. GSEA was performed to compare the expression profiles between high *ACOT8* and low *ACOT8* samples from TCGA database, and four gene sets were significantly enriched in high ACOT8 samples at nominal *p* < 0.05, FDR < 0.25, and |NES| > 1.0 **(A–D)**.

As was shown in the results presented above, *ACOT8* was significantly low expressed in ccRCC samples compared with that in normal kidney samples, whereas the high expression of *ACOT8* represented poor prognosis of ccRCC. These results suggested that ACOT8 may exert anti-tumor effects on ccRCC development but play a role in promoting ccRCC progression. To further explore the functional significance of ACOT8 in ccRCC development and progression, a list of *ACOT8* co-expressed genes in TCGA KIRC dataset with correlation coefficient |*r*| > 0.35 was obtained by UALCAN. Then we performed GO and KEGG pathway analysis on *ACOT8* co-expressed genes. GO analysis revealed that these genes encoded proteins mainly located in mitochondrion and primarily involved in mitochondrial electron transport, a basic event of OXPHOS (Figures 6A–C). KEGG pathway analysis indicated that *ACOT8* co-expressed genes were mainly enriched in OXPHOS pathway, which was consistent with the GO analysis result (Figure 6D). Furthermore, a PPI network was constructed to analyze the genes co-expressed with *ACOT8* in ccRCC (Figure 6E). Then, the top three hub gene clusters were identified by Cytoscape software. GO analysis further indicated that the gene cluster with the highest degree of connectivity was also enriched in mitochondrial electron transport (Figure 6F and Supplementary Figure 6A), while the other two gene clusters were mainly involved in mitochondrial translation and protein ubiquitination (Supplementary Figures 6B,C). To further investigate the functional characteristics of ACOT8 in ccRCC, we performed GSEA on 598 samples (72 normal vs 526 tumor) from TCGA database. The result revealed that genes involved in OXPHOS, fatty acid metabolism and peroxisome assembly, as well as genes up-regulated by reactive oxygen species (ROS) were notably enriched in samples with high *ACOT8* expression (Figures 7A–D). Altogether, these results suggest that ACOT8 may affect OXPHOS through regulating mitochondrial electron transport in ccRCC.

### ACOT8 Is Correlated With *GPX4*, *HIF-2*α, *HILPDA*, and *TAZ* in ccRCC

**FIGURE 8:**
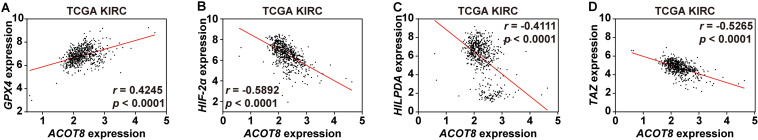
*ACOT8* is correlated with ferroptosis-related genes in ccRCC. Correlation between *ACOT8* and four ferroptosis-related genes in ccRCC was analyzed using TCGA KIRC dataset **(A–D)**.

Interestingly, we noticed that *GPX4*, a central regulator of ferroptosis in ccRCC, existed in the *ACOT8* co-expressed gene list (Yang et al., 2014). To investigate the potential association of ACOT8 with ferroptosis in ccRCC, we utilized three online ccRCC datasets to perform correlation analysis on ferroptosis-related genes which had been publicly reported in ccRCC, including *GPX4*, *HIF-2*α, *HILPDA*, and *TAZ* (Yang et al., 2014, 2019; Zou et al., 2019). Remarkably, *ACOT8* was found to be positively correlated with ferroptosis-suppressing *GPX4* and be negatively correlated with ferroptosis-promoting *HIF-2*α, *HILPDA*, and *TAZ* (Figures 8A–D and Supplementary Figure 7). In summary, ACOT8 may participate in the regulation of ccRCC by ferroptosis inhibition.

## Discussion

Owing to its insidious symptoms in early stage and high mortality in advanced stage, ccRCC has gained great interest in recent years and studies on its biomarkers are more than at any time in the past. Of note, the discoveries of some biomarkers were based on several well-described cellular processes that can affect the proliferation of ccRCC cells. For example, given the regulatory role of autophagy in ccRCC, the researchers focused on autophagy-related genes and finally demonstrated that P4HB is a novel diagnostic and independent prognostic marker for ccRCC via bioinformatics analysis (Xie et al., 2020). Similarly, the fact that dysregulation of cell polarity plays a critical role in ccRCC cell proliferation and apoptosis gave rise to the finding that a decrease of the cell polarity protein PATJ is associated with poorer overall survival in ccRCC patients (Li et al., 2020). ccRCC, however, is not just a disease with aberrant proliferation of kidney cells, being fundamentally a metabolic disorder (Lucarelli et al., 2019). In view of the fact that lipids are the basic structure of cellular membrane and possess key roles in signaling transduction and energy production, fatty acid metabolism reprogramming is obviously one of the most important events in rapidly proliferating cancer cells (Lucarelli et al., 2019). Indeed, long-chain fatty acids were observed to be increased in ccRCC tissues compared to that in adjacent normal kidney tissues through metabolomics analysis (Hakimi et al., 2016), and most of the enzymes involved in β-oxidation were found to be lower in high-grade ccRCC tissues by proteomics analysis (Wettersten et al., 2015). These studies fuel our enthusiasm to find fatty acid metabolism-related biomarkers indicative of ccRCC development and progression.

Acyl-coenzyme A thioesterase family members are pivotal enzymes in mammalian fatty acid metabolism involved in β-oxidation, lipogenesis, signaling transduction, regulation of ion channel opening, and other significant BPs (Kirkby et al., 2010). The study on transgenic knockout mice indicated that ACOT11 suppresses thermogenesis under cold exposure through eliminating endogenous fatty acid oxidation in brown adipose tissue (Okada et al., 2016). According to a recent study, mitochondrial ACOT9 promotes substrate biosynthesis for lipogenesis and hepatic glucose production through directing acetyl-CoA toward the citric acid cycle, thereby aggravating non-alcoholic fatty liver disease (Steensels et al., 2020). Notably, different ACOTs are aberrantly expressed and show discordant regulatory roles in many kinds of malignancies including lung adenocarcinoma (Jung et al., 2013; Jung et al., 2017), hepatocellular carcinoma (Hung et al., 2014; Lu et al., 2019), breast cancer (Jung et al., 2017), gastric adenocarcinoma (Wang et al., 2018), and acute myeloid leukemia (Zhang et al., 2019). For instance, ACOT7 was observed to be highly expressed in lung cancer patients and was found to contribute to cancer development via promoting cell cycle progression (Jung et al., 2017). On the contrary, ACOT12 is significantly decreased in hepatocellular carcinoma tissues, and was found to epigenetically inhibit epithelial-mesenchymal transition through regulating acetyl-CoA expression, thus suppressing tumor metastasis (Lu et al., 2019). Although most ACOTs show abundant expression in kidney, there is not any published research on their expressions and functions in kidney cancer to date. To this end, the current study initially performed *ACOTs* expression analysis in a total of three ccRCC datasets from TCGA and the GEO databases. The results showed that *ACOT1/2/8/11/13* were significantly downregulated in ccRCC tissues compared to that in adjacent non-tumor kidney tissues in all of these datasets, and in particular, the levels of *ACOT11* were markedly reduced in almost every matched normal-tumor pair. Further ROC analysis revealed that the AUC scores of *ACOT1/2/8/13* ranged from 0.7 to 0.9, suggesting their moderate diagnostic values for ccRCC. It was noteworthy that the AUC score of *ACOT11* was up to approximately 0.95, indicating that ACOT11 might be a diagnostic biomarker with extremely high specificity and sensitivity.

Several studies have highlighted prognostic roles of distinct ACOTs in multiple cancers. High expression of *ACOT1* is related to unfavorable prognosis of gastric adenocarcinoma via increased tumor-promoting protein GLI3 (Wang et al., 2018), while *ACOT8* is demonstrated to be an independent predictor of lymph node metastasis and survival of lung adenocarcinoma (Jung et al., 2013). In our work, it seemed that *ACOT1/2/11/13* expressions had no difference during ccRCC progression. Intriguingly, although *ACOT8* was significantly decreased in ccRCC tissues compared with that in normal kidney tissues, its transcriptional expression level kept rising as the tumor progressed. Further clinical correlation analysis revealed that *ACOT8/11* expressions were related to TNM stages of ccRCC, whereas the expression only of *ACOT8* had a significant correlation with histological grades. Consistently, survival curves indicated that higher *ACOT8* expression was associated with worse overall survival of ccRCC, and multivariate Cox regression analysis further validated that *ACOT8* was an independent prognostic marker for ccRCC. All the results above suggested that ACOT8 may play discordant roles in ccRCC tumorigenesis and progression.

The “odd” transcriptional expression pattern of *ACOT8* stimulated our interest in investigating its functional role in ccRCC. The results of GO and KEGG analysis showed that the genes co-expressed with *ACOT8* in ccRCC were primarily involved in mitochondrial electron transport, which is the basis event of OXPHOS. Separately, PPI network analysis indicated that the hub gene cluster was also enriched in the electron transport chain and was mainly composed of genes involved in complex I assembly. GSEA further revealed that genes involved in OXPHOS were enriched in high *ACOT8* samples with the highest | NES| score. As the classical theory of tumor metabolism, Warburg effect reveals that most of cancer cells are dependent on aerobic glycolysis for energy production even under well-oxygenated environment, which leading to the assumption that OXPHOS is downregulated in cancer cells, thereby protecting cells against excessive ROS production (Lucarelli et al., 2019). Consistent with this, mitochondrial respiratory chain has also been proved to be disrupted in ccRCC. As a target gene of HIF-1α, *NDUFA4L2* encodes for a regulatory protein that can block the electron flow from complex I to ubiquinone and is markedly elevated in ccRCC with a prognostic effect on tumor behavior (Tello et al., 2011; Lucarelli et al., 2018). However, recently it has been shown that the difference of mitochondrial DNA content and mutation in different types of cancer cells, as well as the heterogeneity in tumors make it possible for cancer cells to still be highly dependent on OXPHOS under some conditions (Ashton et al., 2018). Interestingly, metabolomics analysis in ccRCC carried out by Hakimi et al. (2016) showed that metabolite levels of OXPHOS are reduced at pathogenesis and elevated during progression, which is consistent with *ACOT8* expression pattern in ccRCC observed in our bioinformatics analysis. Therefore, our results suggested that ACOT8 might positively regulate OXPHOS through electron transport chain in ccRCC. Further experimental evidences are needed to uncover the relationship between ACOT8 and OXPHOS in ccRCC.

In recent years, ferroptosis, an iron-mediated form of non-apoptotic regulated cell death caused by polyunsaturated lipid peroxidation, has been extensively studied in multiple cancers including ccRCC (Bebber et al., 2020). A study in 60 cancer cell lines from eight different tissues showed that ccRCC is particularly susceptible to GPX4-regulated ferroptosis, compared with the other tissues examined (Yang et al., 2014). GPX4 exerts protective roles in cells by using glutathione (GSH) to catalyze the reduction of lipid peroxides, and GPX4 inhibition was observed to be sufficient to kill ccRCC cell lines through ferroptotic cell death (Yang et al., 2014; Stockwell et al., 2020). Several studies further disclosed the association of fatty acid metabolism with ferroptosis in ccRCC. As reported by Miess et al. (2018), the increase in polyunsaturated fatty acids caused by β-oxidation inhibition in ccRCC renders cells highly dependent on the GSH/GPX pathway to withstand ensuing lipid peroxidation and ferroptosis. Intriguingly, as a central oncogenic driver in ccRCC tumorigenesis, HIF-2α was found to selectively enrich polyunsaturated lipids via activating downstream HILPDA, thus conferring sensitivity to ferroptosis (Zou et al., 2019). Moreover, a recent study demonstrated that high ccRCC cell density leads to inactivation of TAZ, one of the central effectors of the Hippo pathway, and subsequently decreases lipid ROS production, thereby abrogating ferroptotic cell death (Yang et al., 2019). In our work, correlation analysis indicated that *ACOT8* expression was positively related to ferroptosis-suppressing *GPX4*, but was negatively correlated with ferroptosis-promoting *HIF-2*α, *HILPDA*, and *TAZ* in a total of three ccRCC datasets from TCGA and the GEO databases. Given that higher *ACOT8* represents shorter overall survival of ccRCC as concluded above, we postulated that ACOT8 might take part in ferroptosis inhibition to regulate tumor progression. Further researches are required to confirm this speculation.

There were several limitations in our work. Most of the data analyzed in this study were obtained from the online databases, and the clinical sample size in our study was small, further large-sample studies are needed to verify our findings. In addition, there was a lack of functional analysis and mechanism exploration by *in vitro* and *in vivo* experiments, so the conclusion we got in this study require further experimental evidences to prove.

## Conclusion

Our study explored the potential roles of ACOTs in the diagnosis and prognosis of ccRCC. According to our analysis, ACOT11 is significantly decreased in ccRCC, showing extremely high values as a diagnostic biomarker, while ACOT8 is an independent prognostic marker for survival outcome. Besides its regulatory roles in fatty acid metabolism, ACOT8 may also affect ccRCC development and progression by the regulation of OXPHOS and ferroptosis. On this basis, further experimental evidences and large-sample clinical studies may uncover the specific mechanisms and bring us one more step nearer to the goal of precise diagnosis and personalized therapy in ccRCC.

## Data Availability Statement

Publicly available datasets were analyzed in this study. This data can be found here: http://portal.gdc.cancer.gov/, http://www.ncbi.nlm.nih.gov/geo/.

## Ethics Statement

The studies involving human participants were reviewed and approved by the Ethics Committee of Shanghai General Hospital, Shanghai Jiao Tong University School of Medicine. The patients/participants provided their written informed consent to participate in this study.

## Author Contributions

M-LS and YS designed and supervised the procedure. LC and DL collected the data. C-LX and F-TC analyzed and visualized the results. DL and F-TC helped to perform the statistical analysis and polish the manuscript. All authors read and approved the final manuscript.

## Conflict of Interest

The authors declare that the research was conducted in the absence of any commercial or financial relationships that could be construed as a potential conflict of interest.

## Footnotes

1 http://ualcan.path.uab.edu/

2 http://portal.gdc.cancer.gov/

3 http://www.ncbi.nlm.nih.gov/geo/

4 http://bioinfo.henu.edu.cn/KIRC/KIRCList.jsp

5 http://david.ncifcrf.gov/

6 http://string-db.org
