# Supplementary material for: Acyl-CoA Thioesterase 8 and 11 as Novel Biomarkers for Clear Cell Renal Cell Carcinoma
Source: Front Genet. 2020 Dec 10;11:594969. doi: 10.3389/fgene.2020.594969 (PMC7758486; doi:10.3389/fgene.2020.594969)
Supplement: Supplementary file 1 [file Data_Sheet_1.docx]

**Table S1.** Primer sequences used in qRT-PCR.

| **Gene** | **Primer sequences (5'-3')** | |
| --- | --- | --- |
| *ACOT1* | Sense | CTGGAGTACTTTGAAGAAGCTGT |
|  | Antisense | AATTCCAAGCAGCCCAACTC |
| *ACOT2* | Sense | CGTCCCGGCTGTACCAATG |
|  | Antisense | GGAACCCTAATGATCTGACCAAC |
| *ACOT8* | Sense | GGCCGCCTATATCTCCGACT |
|  | Antisense | ATGAAGTGCACCTTGTGCTG |
| *ACOT11* | Sense | ATCCAGAATGTCGGAAATCACC |
|  | Antisense | ATCCAGAATGTCGGAAATCACC |
| *ACOT13* | Sense | GGGAGGTGATAAAGGCCATGA |
|  | Antisense | TGCCTATTGCATTGGTATGCTC |
| *GPX4* | Sense | GAGGCAAGACCGAAGTAAACTAC |
|  | Antisense | CCGAACTGGTTACACGGGAA |
| *HIF-2α* | Sense | GGACTTACACAGGTGGAGCTA |
|  | Antisense | TCTCACGAATCTCCTCATGGT |
| *HILPDA* | Sense | AAGCATGTGTTGAACCTCTACC |
|  | Antisense | TGTGTTGGCTAGTTGGCTTCT |
| *TAZ* | Sense | TCCCAGCCAAATCTCGTGATG |
|  | Antisense | AGCGCATTGGGCATACTCAT |

**Table S2.** Association of different *ACOTs* with clinicopathologic characteristics in ccRCC patients.

| **Classification** | **Number of cases** | ***ACOT1*** | | ***p-*Value** | ***ACOT2*** | | ***p-*Value** | ***ACOT13*** | | ***p-*Value** |
| --- | --- | --- | --- | --- | --- | --- | --- | --- | --- | --- |
|  |  | **Low (n=250)** | **High (n=251)** |  | **Low (n=250)** | **High (n=251)** |  | **Low (n=250)** | **High (n=251)** |  |
| **Age (year)** |  |  |  |  |  |  |  |  |  |  |
| ＜60 | 231 (46.1%) | 123 | 108 | 0.166 | 122 | 109 | 0.228 | 111 | 120 | 0.444 |
| ≥60 | 270 (53.9%) | 127 | 143 |  | 128 | 142 |  | 139 | 131 |  |
| **Gender** |  |  |  |  |  |  |  |  |  |  |
| Male | 329 (65.7%) | 171 | 158 | 0.199 | 175 | 154 | *0.042* | 179 | 150 | *0.005* |
| Female | 172 (34.3%) | 79 | 93 |  | 75 | 97 |  | 71 | 101 |  |
| **TNM stage** |  |  |  |  |  |  |  |  |  |  |
| Ⅰ + Ⅱ | 300 (59.9%) | 139 | 161 | 0.051 | 139 | 161 | 0.051 | 141 | 159 | 0.113 |
| Ⅲ + Ⅳ | 201 (40.1%) | 111 | 90 |  | 111 | 90 |  | 109 | 92 |  |
| **Histological grade** |  |  |  |  |  |  |  |  |  |  |
| G1-2 | 226 (45.1%) | 109 | 117 | 0.498 | 107 | 119 | 0.300 | 109 | 117 | 0.498 |
| G3-4 | 275 (54.9%) | 141 | 134 |  | 143 | 132 |  | 141 | 134 |  |

*p-Value less than 0.05 are in italics.*

**Table S3.** Univariate and multivariate Cox regression analyses of overall survival in ccRCC patients.

| **Risk factors** | **Univariate analysis** | | |  | **Multivariate analysis** | | |
| --- | --- | --- | --- | --- | --- | --- | --- |
|  | **HR** | **95% CI** | ***p*-Value** |  | **HR** | **95% CI** | ***p*-Value** |
| Age | 1.804 | 1.291-2.521 | *0.001* |  | 1.589 | 1.132-2.231 | *0.007* |
| Gender | 1.061 | 0.764-1.474 | 0.723 |  |  |  |  |
| TNM stage | 4.241 | 3.006-5.982 | *0.000* |  | 3.276 | 2.277-4.714 | *0.000* |
| Histological grade | 2.826 | 1.945-4.107 | *0.000* |  | 1.877 | 1.268-2.779 | *0.002* |
| *ACOT1* expression | 0.702 | 0.511-0.966 | *0.030* |  | 0.775 | 0.560-1.072 | 0.124 |

*p-Value less than 0.05 are in italics.*
