# Supplementary material for: Acyl-CoA Thioesterase 8 and 11 as Novel Biomarkers for Clear Cell Renal Cell Carcinoma
Source: Front Genet. 2020 Dec 10;11:594969. doi: 10.3389/fgene.2020.594969 (PMC7758486; doi:10.3389/fgene.2020.594969)
Supplement: Supplementary file 2 [file Presentation_1.pdf]

Supplementary Material

Supplementary Figures

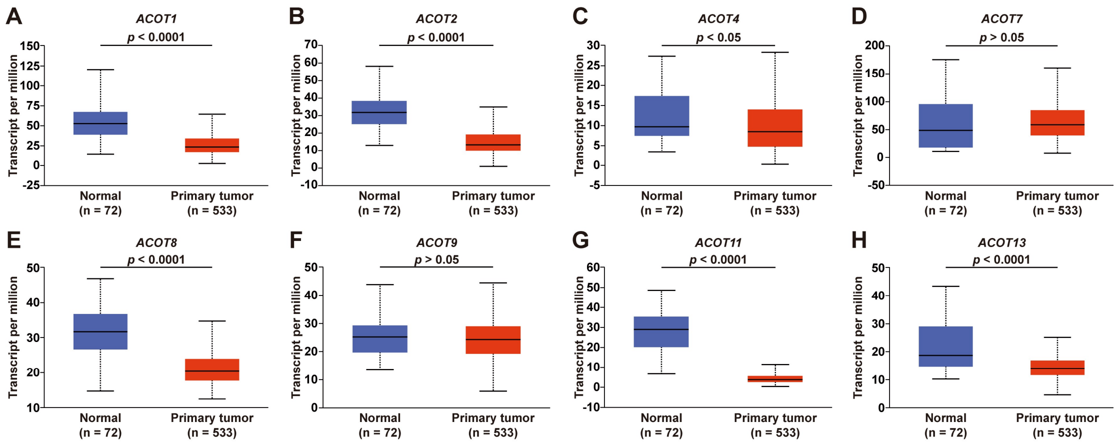

Figure S1. Different *ACOTs* are downregulated in ccRCC samples. The transcriptional expressions of different *ACOTs* in ccRCC and normal kidney samples were analyzed by UALCAN. *ACOT1/2/4/8/11/13* were significantly low expressed in ccRCC, while the two groups had no difference of significance in *ACOT7/9* expressions (**A-H**). p value < 0.05 was regarded as statistically significant.

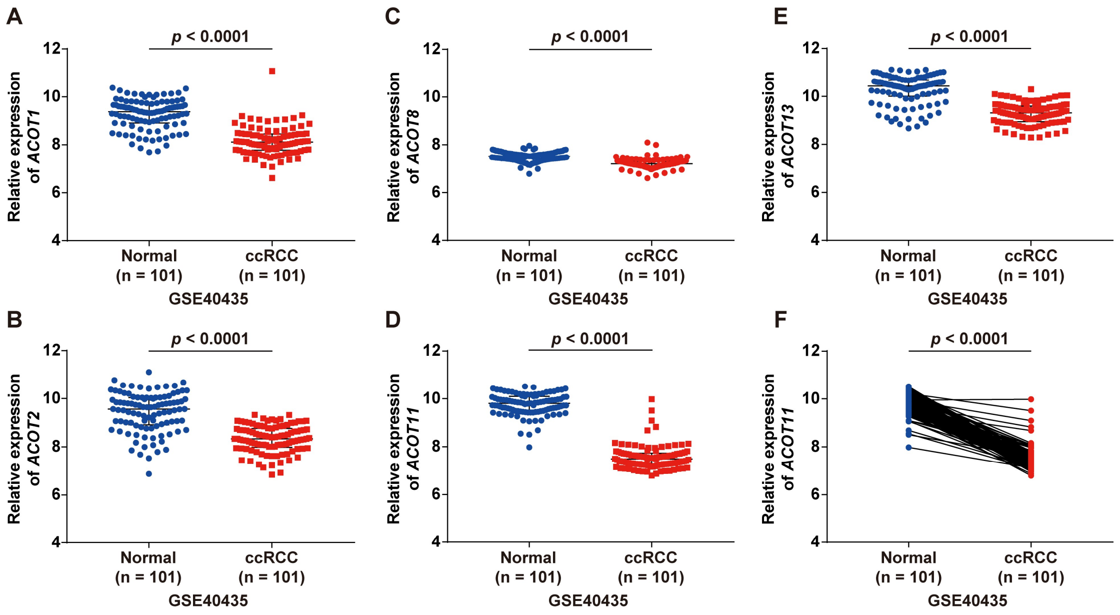

Figure S2. Different *ACOTs* expressions are verified in 101 ccRCC normal-tumor pairs from GSE40435. *ACOT1/2/8/11/13* were significantly low expressed in ccRCC samples from GSE40435 (**A-E**). *ACOT11* expression significantly decreased in almost every matched sample (**F**). p value < 0.05 was regarded as statistically significant.

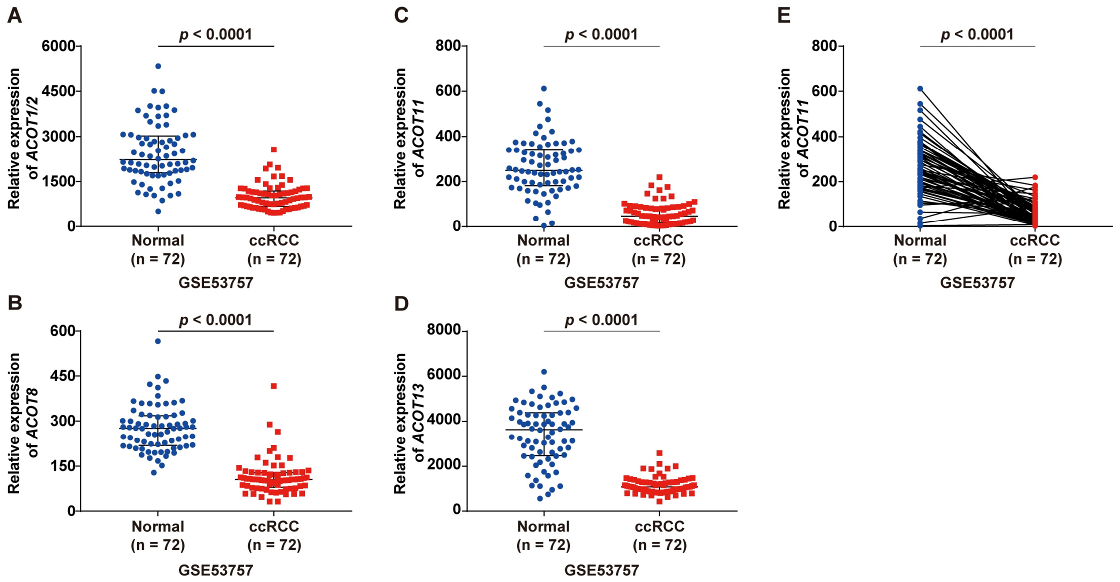

Figure S3. Different *ACOTs* expressions are validated in 72 ccRCC normal-tumor pairs from GSE53757. *ACOT1/2/8/11/13* expression were obviously downregulated in matched ccRCC samples from GSE53757 (**A-D**). *ACOT11* were markedly low expressed in most paired samples (**E**). p value < 0.05 was considered as statistically significant.

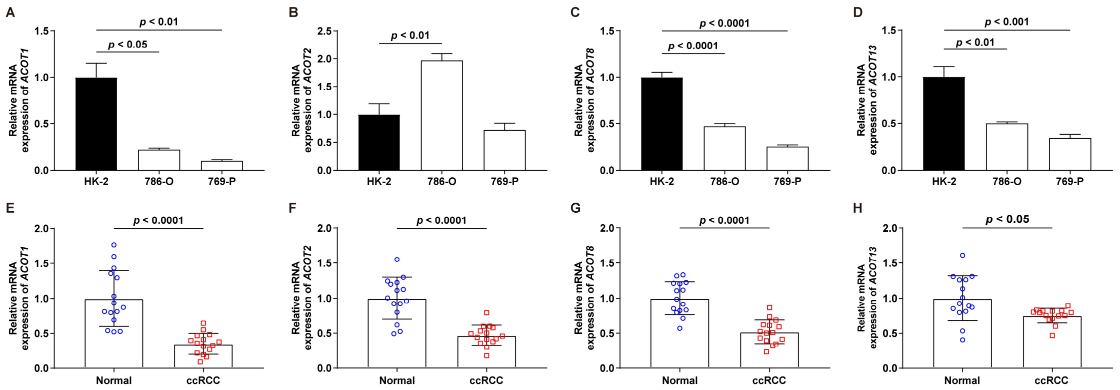

Figure S4. Validation of different *ACOTs* expressions in ccRCC. The mRNA expressions of *ACOT1/2/8/13* were measured in ccRCC cell lines (**A-D**) and clinical samples (**E-H**) by qRT-PCR. p value < 0.05 was considered to be statistically significant.

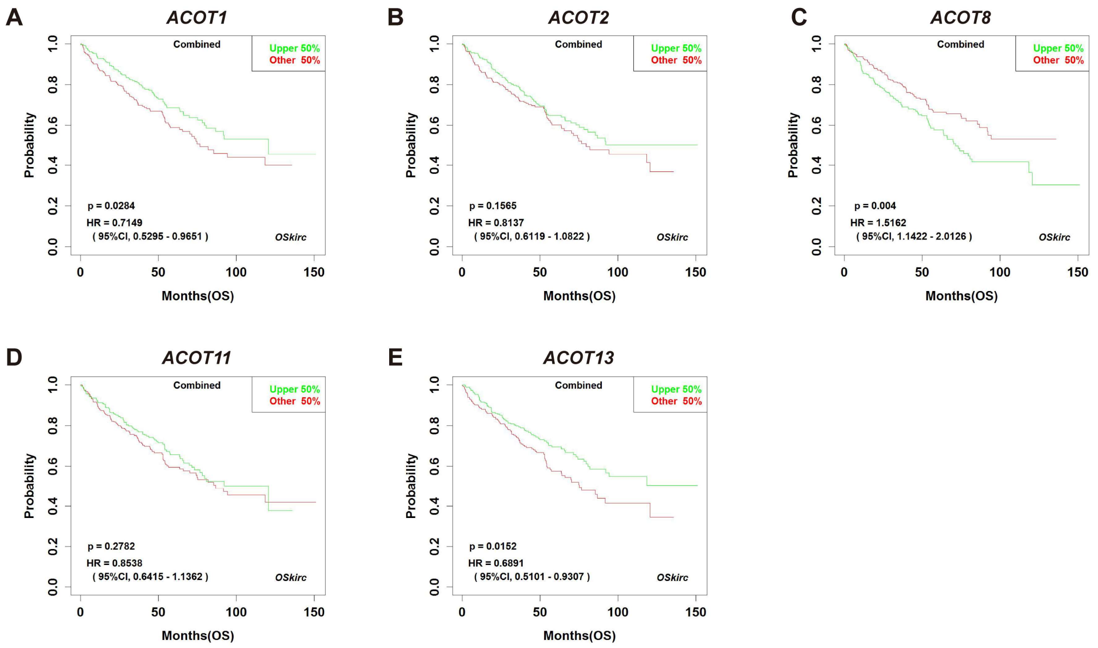

Figure S5. Survival analysis of different *ACOTs* in ccRCC. ccRCC cases (n=629) with clinical follow-up information were utilized for survival analysis of different *ACOTs* by OSkirc. *ACOT1/8/13* expressions were significantly associated with the overall survival in ccRCC patients (**A, C** and **E**), while the mRNA expressions of *ACOT2/11* had no correlation with the prognosis of ccRCC patients (**B**, **D**). Log-rank p value < 0.05 was considered to be statistically significant.

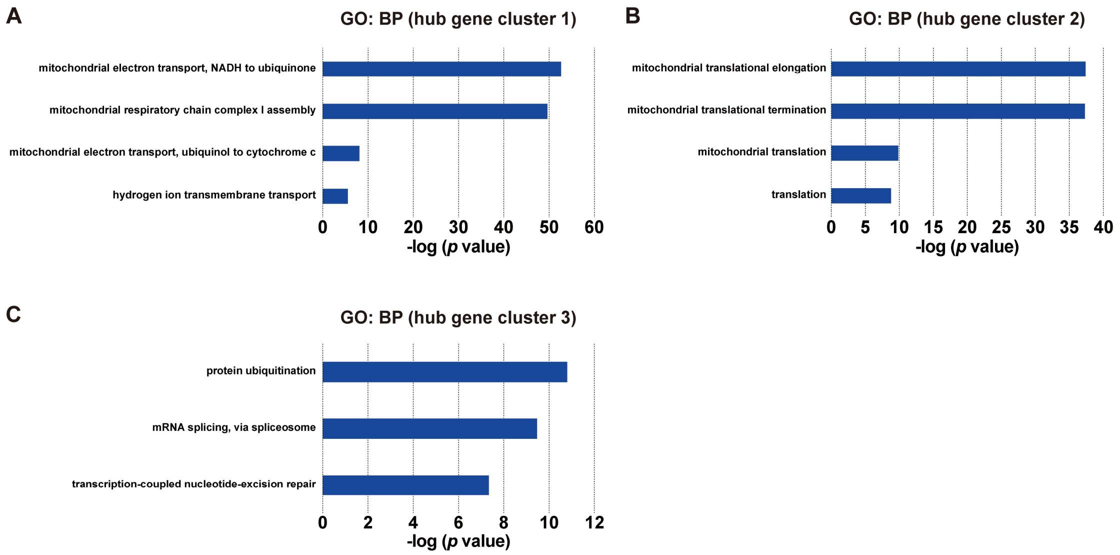

Figure S6. GO analysis on the top 3 hub gene clusters of *ACOT8* co-expressed genes. GO biological process (BP) analysis was performed on the top three hub gene clusters of *ACOT8* co-expressed genes. The hub gene cluster with the highest score of connectivity was primarily enriched in mitochondrial electron transport (**A**). The second hub gene cluster was mainly involved in mitochondrial translation (**B**) and the third hub gene cluster was primarily enriched in protein ubiquitination (**C**).

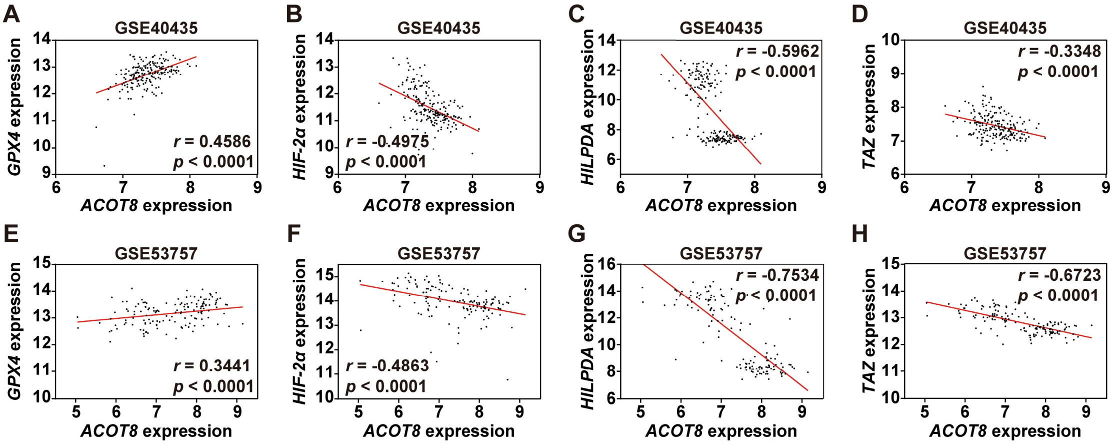

Figure S7. *ACOT8* is correlated with ferroptosis-related genes in ccRCC. Correlation between *ACOT8* and four ferroptosis-related genes in ccRCC was analyzed using two ccRCC datasets from the GEO database (GSE40435 and GSE53757).
